# Mood, Burnout, and Dispositional Optimism in Kayak Polo Players During Their Competitive Stage

**DOI:** 10.3389/fpsyg.2021.667603

**Published:** 2021-05-21

**Authors:** Salvador Angosto, Laura Salmerón-Baños, Francisco José Ortín-Montero, Vicente Morales-Baños, Francisco José Borrego-Balsalobre

**Affiliations:** ^1^Faculty of Sport Science, Sport and Physical Activity, CEI Mare Nostrum, University of Murcia, San Javier, Spain; ^2^Faculty of Psychology, Basic Psychology and Methodology, CEI Mare Nostrum, University of Murcia, Murcia, Spain

**Keywords:** psychological variables, sport performance, training, IBD-R, LOT-R, POMS, kayak

## Abstract

The main objective of performance sport is to obtain achievements at the highest level through the adequate development of the athlete. The scientific literature demonstrates the fundamental role played by the inclusion of certain psychological variables in the training plan. This study examined the psychological profile of kayak polo players through the variables of burnout, optimism, and mood in the hours prior to the competition, relating these to each other and to some sociodemographic data. A sample of 86 canoeists, 60 men (69.8%) and 26 women (30.2%), with an age measurement of 24.4 ± 9.1 years belonging to the first male and female kayak polo division, completed the POMS-29, the LOT-R, and the IBD-R. Athletes' levels of optimism were found to be significantly correlated with mood. Optimism also influenced emotional exhaustion. In addition, seniority and internationality were decisive factors in the level of optimism and mood achieved.

## Introduction

High-level competitive sport is understood as a sport whose aim is for athletes to perform at their best. However, reaching the peak of an athlete's performance and maintaining it at the highest level for as long as possible is not an easy task, since, in addition to taking into consideration physical, technical, tactical, and strategic resources, it is necessary to introduce psychological variables into the formula for sporting success (Gould and Maynard, 2009; Mauss and Robinson, 2009; Barker et al., 2020). Many specialists conclude that competitive sport involves the intervention and inherent control of aspects such as motivation, attention, stress, anxiety, self-confidence, moods, self-control and self-regulation, cohesion, interpersonal skills, or emotional adjustment (Meyers et al., 1979; Garcia Quinteiro et al., 2006; Gimeno et al., 2007; Almagro et al., 2020).

Mastering and correctly training all these variables can contribute to the optimization of sporting performance, increasing the possibilities of improving results, and at the same time ensuring continuity in competitive sport for a prolonged period of time. In this sense, in order to find out the optimal relationship between the different psychological variables and their consequent positive effect on sporting results, numerous studies have focused their research on the analysis of these variables, sporting experiences, and the success achieved in the results (Kreinerphillips and Orlick, 1993; Vernacchia et al., 2000; Gould et al., 2002).

It is necessary to be meticulous with the training work carried out by athletes, in view of the fact that sports practice, in the first instance, is associated with pleasant effects for most of them. However, working with high training loads and the competition itself can lead to a situation of chronic stress, which eventually results in the need for abandonment (Gustafsson et al., 2008, 2018). Intense and prolonged sporting efforts, accompanied by sporting failures, may be susceptible to sport dropout (Lemyre et al., 2008). Not achieving the expected results or not reaching the proposed objectives leads athletes to a situation of exhaustion, both physically and psychologically, giving rise to negative thoughts about their abilities to practice the sport (Raglin, 2001).

Having established the above provisions, it is therefore easy to conclude that, in the competitive field, it is necessary to carry out a psychological evaluation of the athlete, in order to be able to establish an objective and individualized assessment of the variables that may be most influential in each case. To this end, analytical tools such as questionnaires are required, which, together with other elements and instruments for recording and collecting data, can provide valuable and decisive information for the training of the athlete, as well as for their training for competition (Gimeno et al., 2007). In line with what has been described, the literature on the analysis of psychological variables, as well as the variety and typology of questionnaires for such analysis, is extensive (López Roel and Dosil, 2019); this has undergone a significant evolution in recent decades, from the use of more generic measurement instruments to the development and use of more specific instruments for sports (Biddle, 1992; Hall et al., 1998; Anshel and Sutarso, 2007; Fayos Ruiz et al., 2012; Andrade et al., 2013; Cano-García et al., 2015).

### Burnout Syndrome

Having described the statements in the previous paragraphs, it is necessary to specify that taking into account the burnout of the athlete as a result of training overload is to consider a multidimensional phenomenon where different factors come into play, such as emotional and physical fatigue, decreased achievement, and sport devaluation (Goodger et al., 2007a; Reche et al., 2018). Early studies on burnout defined the concept within the framework of feelings of failure and exhaustion in the face of excessive demands on energy, strength, and personal resources (Carlin and Garcés de los Fayos Ruiz, 2010). Other works highlighted emotional exhaustion, depersonalization, and low self-fulfillment (Jackson and Maslach, 1982; Byrne, 1993).

Furthermore, other researchers added that, in sports, the hours of training and the competition itself could be a source of chronic psychological stress, which was significantly related to the risk of burnout and abandonment of sports practice (Hall et al., 1998; Hill et al., 2010). This abandonment is qualified in certain studies as one of the most worrying effects of the syndrome for professional athletes (García-Parra et al., 2016), not only because of the psychological consequences but also because of the social consequences, which may involve a questioning of the identity acquired during their time as athletes (Coakley, 1992; Goodger et al., 2007a). Along these lines, athletes experiencing burnout have common characteristics, but the symptoms manifested may be completely different (Gustafsson et al., 2018). This highlights the need to develop analysis strategies through tools that allow personalizing and professionally treating athletes with individualized work plans.

In the context of sport, there are four fundamental lines of work in burnout research. First, the identification of predictor variables. Second, work aimed at analyzing the consequences of suffering from the syndrome. As a third line of research, the authors point to the development of models that help to better understand the syndrome. Finally, there is a line of research that collects epidemiological data allowing us to analyze the incidence of this syndrome in the context of sport (Carlin and Garcés de los Fayos Ruiz, 2010; García-Parra et al., 2016).

Thus, in the last decade, numerous studies can be found in which the authors measure burnout syndrome in different collective sports modalities such as football (Lopes Verardi et al., 2015), rugby (Cresswell and Eklund, 2005, 2006), and volleyball (Vieira et al., 2013). On the other hand, research on burnout in individual sports such as athletics (Reche et al., 2018), swimming (Larson et al., 2019), tennis and table tennis (Goodger et al., 2007b; Martinent et al., 2014, 2020), or olympic wrestling (Gil et al., 2015) stand out.

### Dispositional Optimism

The scientific literature provides information on the relationship between the dimensions of burnout syndrome and a more or less optimistic profile (Chen et al., 2008; García et al., 2014; Gil et al., 2015). Throughout their lives, human beings face a wide variety of situations, solving them with very different attitudes and actions. In this sense, the existence of two very different styles of resolution, optimistic and pessimistic, is postulated through the theory of explanatory patterns, where people give explanations to the events that happen to them (Abramson et al., 1978; Herzberg et al., 2006). In contrast, other researches defend another perspective, through dispositional theory, defined as the stable expectation or belief that positive things will happen in life, known as dispositional optimism (Scheier and Carver, 1987; Andersson, 2012).

Optimism is a cognitive construct related to motivation that reflects the degree to which people have generalized favorable expectations for their future. Higher levels of optimism have been prospectively related to better subjective well-being in times of adversity or hardship (Carver et al., 2010). The study of optimism was largely initiated in health contexts, finding positive associations between optimism and markers of better physical and psychological health. More recently, the scientific study of optimism shows that optimists have better social connections (Brissette et al., 2002; Carver and Scheier, 2014).

In line with what has been established, certain studies on sport confirm that optimism is negatively related to all the dimensions of burnout, so that as optimism increases, the scores in the different dimensions of burnout decrease. On the other hand, optimism is presented as a variable associated mainly with regular, continuous, and regulated sports practice, since the highest scores are found in older, federated athletes who compete and spend more hours per week and years of dedication to sports practice (Andersson, 1996; Gould et al., 2002; García et al., 2014).

In this line, there have been numerous investigations developed in recent years in relation to collective sports concerning optimism in the disciplines of football (Chirivella et al., 2013), basketball (Gordon, 2008), and handball (Ortin-Montero et al., 2013) among other sports modalities. They have also been developed in relation to individual sports, in disciplines such as tennis (Gustafsson and Skoog, 2012), swimming (Norlander and Archer, 2002), or athletics (Vaamonde, 2018).

### Athlete's State of Mind

Having defined the variables of burnout and dispositional optimism, it is necessary to qualify that both can influence the mood with which people face different situations (Remor et al., 2006; Kim et al., 2009; Grobbelaar et al., 2010; Remor and Gómez, 2013; Sánchez-Hernández et al., 2014). Scientific evidence suggests that optimism can protect health at times of increased stress by influencing mood (Brydon et al., 2009). Precisely in relation to mood, it can be stated that a person suffering burnout syndrome is apathetic, easily irritable, with low frustration tolerance, and recurrent thoughts of incompetence. This can lead to absenteeism, the onset of addictive behaviors, and it can affect the social aspect by generating susceptibility to interpersonal contact. It is highly probable that, as a consequence, a person with burnout syndrome becomes socially isolated, as a functional interpersonal strategy, possibly facilitating the presence of mood and anxiety disorders, which could undoubtedly affect not only their work performance and development but also their personal development (Schaufeli et al., 1993).

In physical sporting activity, the emotional component acquires a multidimensional nature. Its study, knowledge, and treatment allow us to obtain a better approach to the reality of the athlete and to improve both their assessment and the design of appropriate training strategies. The athlete, throughout his activity and sporting life, presents different states, each of which is the result of various factors or variables. In the relationship between mood and performance in sport, one of the most relevant results in the sport psychology field refers to the perceptions that athletes have about their own moods, which can somehow influence their performance (Arce Fernández et al., 2000; de la Vega Marcos et al., 2008; Antonio Arruza et al., 2011). The importance of knowing the level of stability of moods lies in the need to offer specific guidelines to the professional in charge of achieving the maximum performance of the athlete, allowing him/her, first, to predict where more complications may arise in the competitive coping of sport and, second, to be able to intervene in order to optimize and benefit the athlete's performance (Hassmen and Blomstrand, 1995; Rietjens et al., 2005).

In the same way as for the variables of burnout and dispositional optimism, in the literature, there are multiple investigations carried out through measurement tools for the analysis of moods of athletes in collective disciplines such as basketball (Henderson et al., 1998; Hoffman et al., 1999) and football (Andrade et al., 2008; Saidi et al., 2020) or individual disciplines such as swimming (Santhiago et al., 2011) and cycling (Viana et al., 2016), among others. Hence, researching burnout, optimism, and moods together may be of interest, insofar as there are studies of these variables paired or separately, but not all three together, for the purpose of developing a psychological profile of the athlete as appropriate as possible and planning the work according to the demands of the training loads or even the competition itself.

### Effects of Psychological Variables in Kayak Polo

Canoeing is a water sport in continuous evolution as a result of the emergence of new disciplines and modalities adapted to the originated demand, forcing to establish, in the field of competition, new methods and training systems, sensitive to the progress of research in the field (Zarodnyuk et al., 2019). These disciplines are mostly of an individual nature, although there are also those of a collective nature. Competitions, as a general rule, take place over several days, since it is common for many athletes to participate in more than one category (individual K1/C1, and in team boats, K2–K4/C2–C4) causing a greater workload and possible stress on the athlete. Specifically, kayak polo, as the only collective discipline of canoeing where several individual boats take part at the same time in the same team, also often presents competition systems by means of gatherings, tournaments, or scoring days of several matches, where the demands are high.

Taking into account the inequality of disciplines, as well as the typology of efforts in competition, canoeing can be classified as an endurance sport, in which good aerobic capacity, aerobic efficiency at the anaerobic threshold, and lactic anaerobic capacity, especially lactate tolerance, are required to obtain a good performance (Fry and Morton, 1991; Faina et al., 1997). Therefore, canoeing is a sport where strength and endurance are developed together to optimize the athlete's performance (Bishop et al., 2002; Garcia-Pallares et al., 2009), factors that entail a greater training load, which can have an impact on the psychological aspect of athletes (Isorna Folgar et al., 2019).

The present study arises from the need to delve a little deeper into kayak polo, a minority sport, since there are few empirical studies carried out with this discipline. The limited number of research studies found deals with anthropometric (Rodrigues Alves et al., 2012), physiological (Rodrigues Alves et al., 2012; Forbes et al., 2013; Sheykhlouvand et al., 2018; Zwingmann et al., 2020), and psychological (Hill et al., 2010) aspects of sport performance. This scarcity of specific studies on psychology in this sport makes it even more interesting to develop studies on the subject. In this sense, the aim of this study is to evaluate the psychological profile of kayak polo players throughout three variables of burnout, dispositional optimism, and mood in the hours prior to a national and international competition, also relating these aspects with some sociodemographic data. The instruments used for this research are the Spanish version of the POMS-29 mood scale (Balaguer et al., 1993), the Spanish version of the Life Orientation Test Revised LOT-R (Cano-García et al., 2015), and the Inventory of Burnout in Athletes Revised IBD-R (Fayos Ruiz et al., 2012).

The analysis of these variables can provide valuable information on trait and state aspects of kayak polo players when facing potential stressful situations, as well as contribute to the planning of future intervention through psychological skill training based on the outcome of the assessments and the establishment of the relationship between the constructs and variables assessed in order to optimize performance.

## Materials and Methods

### Sample

Table 1 shows the descriptive statistics of the sample. The sample was composed of a total of 86 kayak polo players, 69.8% were male and 30.2% female with a mean age of 24.4 ± 9.1 years, who participated in the First Division male and female Spanish league. Regarding the category to which the athlete belonged, 60.5% were seniors, 39.5% were U21, and 44.2% were or had been international players. The majority of the players played in the defensive position of winger (44.2%), followed by the advanced and central positions with 24.4 and 17.4%, respectively. Goalkeeper was the least represented position with 14.0%. Approximately half of the players trained 5 days or more per week (48.8%), followed by those who trained 4 days (22.1%) and 3 days (18.6%). Regarding training hours, 44.2% spent more than 10 h per week and 34.9% trained between 6 and 10 h per week.

**Table 1:** Sociodemographics statistics of the sample.

	**N**	**%**
**Gender**
Male	60	69.8
Female	26	30.2
**Category**
Under21	34	39.5
Senior	52	60.5
**National team member**
No	48	55.8
Yes	38	44.2
**Defensive player position**
Advanced	21	24.4
Central	15	17.4
Winger	38	44.2
Goalkeeper	12	14
**Weekly sports practice days**
1 day	3	3.5
2 days	6	7
3 days	16	18.6
4 days	19	22.1
5 or more days	42	48.8
**Weekly sports practice time**
0–3 h	3	3.5
3–6 h	15	17.4
6–10 h	30	34.9
10 h or more	38	44.2

### Instruments

The optimism construct was assessed with the Spanish version of the LOT-R questionnaire (Cano-García et al., 2015). The LOT-R is a revision of the Life Orientation Test (Scheier and Carver, 1985), carried out to distinguish optimism from neuroticism (Scheier et al., 1994). In this sense, some authors carried out a psychometric analysis of the revised test, obtaining very similar properties with those of the original version. It is composed of 10 items using a five-point scale, where 0 means “strongly disagree” and 4 means “strongly agree” (Ferrando et al., 2002). Regarding the correctness and interpretation of the test, two options appear: On the one hand, the measurement of each disposition separately, and, on the other hand, the measurement of total optimism by reversing the items written in a negative sense. In this study, we decided to use the second option in line with previous work in the literature (Mroczek et al., 1993; Myers and Steed, 1999).

### Procedure

The data collection was carried out by an online survey using Google Forms. The distribution was carried out by sending an email to the different kayak clubs informing the purpose of the study and with the link to the questionnaire. The clubs that agreed with the study distributed the questionnaire among their athletes. Each athlete responded voluntarily and anonymously. Data collection was conducted before the end of the season between October and November 2016.

### Data Analysis

The statistical program SPSS v.24.0 (IBM, Armonk, NY, USA) was used for data analysis. Descriptive statistics and correlation analysis of the different items and Cronbach's alpha index (C-a) were calculated. Then, a cluster analysis was carried out to identify possible groups of athletes with similar optimism level, taking as a dependent variable, the item “Optimism.” To obtain the cluster solutions, two methods were combined, hierarchical and non-hierarchical, with the aim of optimizing results. The cluster analyses were carried out using the guidelines proposed for computer programs (Milligan, 1985). The hierarchical cluster was analyzed taking the Ward's method, and for the similarity measures, the Euclidean distance squared was used. Then, a non-hierarchical cluster was done through the K-means method, taking as a reference the centroids of the cluster solutions of the hierarchical method for each period. Chi-square tests for qualitative variables and ANOVA test for quantitative variables were performed (Khalilzadeh and Tasci, 2017). Finally, a MANOVA test was carried out (Chapman, 2018), to analyze the relationship between the psychological variables (optimism, mood state, and burnout) as dependent variables, and the independent variables, if the athletes were a member of national team (yes/no) and the time spent training (<10 h a week/more than 10 h a week). The effect size was calculated according to the guidelines of the literature (Dominguez-Lara, 2018). The significance level was established at a value of *p* ≤ 0.05.

## Results

### Descriptive and Correlation Analysis

The general descriptive results of the psychological variables (Table 2) showed that the overall level of optimism of the kayak polo players was moderate (*M* = 2.76 ± 0.6). The mood state profile indicated that they had high vigor (*M* = 2.82 ± 0.6), medium levels of tension, fatigue, and anger, and low levels of depression (*M* = 0.68 ± 0.7). In terms of burnout factors, the players had high values for reduced efficacy (*M* = 3.83 ± 0.6) and low depersonalization (*M* = 1.89 ± 0.6). Correlation analysis (Table 2) indicated that optimism alone was statistically significantly related to the mood states of anger and depression and the emotional exhaustion of burnout. Mood states and burnout dimensions correlated significantly with each other on all dimensions except the relation between tension and vigor.

**Table 2:** Descriptive and correlation analysis.

	**M (SD)**	**1**	**2**	**3**	**4**	**5**	**6**	**7**	**8**	**9**
1. Optimism	2.75 (0.6)	1								
2. Anger	1.18 (1.0)	−0.241^*^	1							
3. Fatigue	1.21 (0.9)	−0.115	0.445^**^	1						
4. Tension	1.86 (0.8)	−0.206	0.461^**^	0.285^**^	1					
5. Depression	0.68 (0.7)	−0.342^**^	0.674^**^	0.425^**^	0.557^**^	1				
6. Vigor	2.82 (0.6)	0.131	−0.333^**^	−0.419^**^	−0.002	−0.364^**^	1			
7. Emotional exhaustion	2.26 (0.8)	−0.231^*^	0.485^**^	0.569^**^	0.238^*^	0.424^**^	−0.446^**^	1		
8. Despersonalization	1.89 (0.6)	−0.100	0.397^**^	0.357^**^	0.337^**^	0.258^*^	−0.303^**^	0.538^**^	1	
9. Reduced efficacy	3.83 (0.6)	0.116	−0.310^**^	−0.390^**^	−0.212^*^	−0.346^**^	0.433^**^	−0.602^**^	−0.352^**^	1

* *p ≤ 0.05*;

** *p ≤ 0.01*.

### Cluster Analysis

#### Group Identification

The cluster analysis was determined according to the variable of optimism (Table 3). Cluster 1, labeled “High Optismism,” made up 27.9% of the sample and represented the athletes who showed the high levels of optimism (*M* = 3.49 ± 0.3). Cluster 2 was designated “Moderate Optimism” because the level of optimism had middle scores (*M* = 2.71 ± 0.2) shown by athletes, represented 50.0% of the sample; and cluster 3, designated “Lower Optimism,” represented 22.1% of the athletes who showed the lower level of optimism (*M* = 1.92 ± 0.4). The mood state profile was similar in cluster 1 and cluster 2; they had high scores in vigor, moderate-lower scores in tension, fatigue, and anger, and lower scores in depression. Cluster 3 had a lower level of vigor than the other groups, moderate-high scores in tension, and moderate-lower scores in the other states. Burnout scores were similar in the three groups with high scores in reduced efficacy, moderate scores in emotional exhaustion, and lower scores in depersonalization. ANOVA test showed statistically significant differences in optimism between all groups, depression between cluster 1 “High Optimism” and cluster 3 “Low Optimism,” and emotional exhaustion between cluster 2 “Moderate Optimism” and cluster 3 “Low Optimism” (*p* ≤ *0.05*).

**Table 3:** Average scores for each variable in the three clusters.

	**High optimism (*n* = 24)**	**Moderate optimism (*n* = 43)**	**Low optimism (*n* = 19)**	**F(df)**	***p*-value**	***$\eta_{p}^{2}$***
	**M (SD)**	**M (SD)**	**M (SD)**			
Optimism^*^	3.49 (0.3)	2.71 (0.2)	1.92 (0.4)	169.19 (2)	0.001	0.80
Anger	1.12 (1.1)	1.03 (0.8)	1.62 (1.1)	2.69 (2)	0.074	0.06
Fatigue	1.18 (1.1)	1.20 (0.7)	1.29 (0.9)	0.09 (2)	0.911	0.00
Tension	1.76 (0.9)	1.85 (0.8)	2.04 (0.6)	0.65 (2)	0.523	0.02
Depression^#^	0.52 (0.8)	0.62 (0.6)	1.05 (0.9)	3.22 (2)	0.045	0.07
Vigor	2.73 (0.8)	2.95 (0.6)	2.67 (0.6)	1.84 (2)	0.166	0.04
Emotional exhaustion^$^	2.27 (1.0)	2.08 (0.6)	2.64 (0.8)	3.52 (2)	0.034	0.08
Despersonalization	1.98 (0.9)	1.79 (0.5)	2.01 (0.4)	1.11 (2)	0.333	0.03
Reduced efficacy	3.83 (0.7)	3.90 (0.5)	3.66 (0.6)	1.06 (2)	0.353	0.03

* *Differences between all groups*.

# *Differences between group 1 and group 3*.

$ *Differences between group 2 and group 3. $\eta_{\text{p},}^{2}$ partial squared eta; between $\eta_{\text{p}}^{2}$ < 0.01 trivial effect, between 0.01 < $\eta_{\text{p}}^{2}$ < 0.06 small effect, between 0.06 < $\eta_{\text{p}}^{2}$ < 0.14 medium effect, and $\eta_{\text{p}}^{2}$ > 0.14 significant effect*.

#### Profile of the Groups

Table 4 shows the sociodemographic profile of the athletes according to the cluster group to which they belong depending on their optimism level. Cluster 1 “High Optimism” were males with an average age of 27.5 ± 10.4 years, who belonged to the senior category; half had been international players for some time and played in the winger defensive position. They went 5 or more days to training in a week and spent between 6 and 10 h of training per week. Cluster 2 “Moderate Optimism” was composed of males with an age of 23.0 ± 8.4 years old, belonged to the Under21 category; the majority two thirds have not been international players, and half plays in winger defensive position. Sports habits indicated that almost half were training 5 or more days a week with more than 10 h of training per week. Finally, cluster 3 “Low Optimism” was made up of males with an average age of 23.6 ± 8.5 years, belonging to the senior category and playing in the position of goalkeeper. Sports habits showed that they trained 5 days or more per week with a training time of more than 10 h per week. There were statistically significant differences in the category and defensive player position variables (*p* ≤ 0.05), while the effect of the variables was low in category position (V < 0.3) and medium effect in the defensive position (V > 0.3).

**Table 4:** Characteristics of the different cluster groups.

	**High optimism (*n* = 24)**	**Moderate optimism (*n* = 43)**	**Low optimism (*n* = 19)**	**F(df)**	***p*-value**	***$\eta_{p}^{2}$***
	**M(SD)**	**M(SD)**	**M(SD)**			
**Age**	27.5 (10.4)	23.0 (8.4)	23.6 (8.5)	2.04(2)	0.136	0.05
	**N (%)**	**N (%)**	**N (%)**	***X**^**2**^**(df)***	***p-*****value**	***V***
**Gender**						
Male	17 (70.8)	28 (65.1)	15 (78.9)	1.21 (2)	0.545	0.12
Female	7 (29.2)	15 (34.9)	4 (21.1)			
Category^*^						
Under21	5 (20.8)	22 (51.2)	7 (36.8)	6.00 (2)	0.050	0.26
Senior	19 (79.2)	21 (48.8)	12 (63.2)			
**National team member**						
No	12 (50.0)	29 (67.4)	7 (36.8)	5.46 (2)	0.065	0.25
Yes	12 (50.0)	14 (32.6)	12 (63.2)			
Defensive player position^*^						
Advanced	7 (29.2)	11 (25.6)	3 (15.8)	18.30 (6)	0.006	0.33
Central	3 (12.5)	8 (18.6)	4 (21.1)			
Winger	12 (50.0)	22 (51.2)	4 (21.1)			
Goalkeeper	2 (8.3)	2 (4.7)	8 (42.1)			
**Weekly sports practice days**						
1 day	1 (4.2)	1 (2.3)	1 (5.3)	2.95 (8)	0.938	0.13
2 days	1 (4.2)	4 (9.3)	1 (5.3)			
3 days	6 (25.0)	8 (18.6)	2 (10.5)			
4 days	6 (25.0)	9 (20.9)	4 (21.1)			
5 or more days	10 (41.7)	21 (48.8)	11 (57.9)			
**Weekly sports practice time**						
0–3 h	2 (8.3)	1 (2.3)	-	8.91 (6)	0.179	0.23
3–6 h	2 (8.3)	9 (20.9)	4 (21.1)			
6–10 h	11 (45.8)	16 (37.2)	3 (15.8)			
10 h or more	9 (37.5)	17 (39.5)	12 (63.2)			

* *p ≤ 0.05. $\eta_{\text{p},}^{2}$ partial squared eta; between $\eta_{\text{p}}^{2}$ < 0.01 trivial effect, between 0.01 < $\eta_{\text{p}}^{2}$ < 0.06 small effect, between 0.06 < $\eta_{\text{p}}^{2}$ < 0.14 medium effect, and $\eta_{\text{p}}^{2}$ > 0.14 significant effect. V, Cramer's V; V < 0,10: irrelevant effect, between 0.10 < V < 0.30: small effect, between 0.30 < V < 0.50: moderate effect, and V > 0.50 large effect*.

### MANOVA Test

MANOVA test (Table 5) showed that the independent variables “National team member” and “Weekly sports practice time” statistically significantly affected the combination of correlated dependent variables of psychological variables of optimism, mood state, and burnout (Pillai's Trace: 0.215, *F* = 2.25, *p* ≤ 0.05, $\eta_{\text{p}}^{2}$ = 0.22; Wilks' lambda: 0.785, *F* = 2.25, *p* ≤ 0.05, $\eta_{\text{p}}^{2}$ = 0.22). The effects between subjects were statistically significant in optimism (*F* = 7.03; *p* ≤ 0.01) and the mood state of anger (*F* = 4.26; *p* ≤ 0.05). The parameter estimates between subjects were significant in all variables, such as reduced efficacy (β = 3.66; t = 30.59; *p* ≤ 0.001; $\eta_{\text{p}}^{2}$ = 0.92), optimism (β = 2.76; *t* = 23.13; *p* ≤ 0.001; $\eta_{\text{p}}^{2}$ = 0.87), vigor (β = 2.73; *t* = 22.02; *p* ≤ 0.001; $\eta_{\text{p}}^{2}$ = 0.86), emotional exhaustion (β = 2.46; *t* = 15.64; *p* ≤ 0.001; $\eta_{\text{p}}^{2}$ = 0.75), depersonalization (β = 2.12; *t* = 16.61; *p* ≤ 0.001; $\eta_{\text{p}}^{2}$ = 0.77), tension (β = 1.83; *t* = 11.50; *p* ≤ 0.001; $\eta_{\text{p}}^{2}$ = 0.62), fatigue (β = 1.14; *t* = 6.37; *p* ≤ 0.001; $\eta_{\text{p}}^{2}$ = 0.33), anger (β = 1.07; *t* = 5.61; *p* ≤ 0.001; $\eta_{\text{p}}^{2}$ = 0.28), and depression (β = 0.69; *t* = 4.62; *p* ≤ 0.001; $\eta_{\text{p}}^{2}$ = 0.21).

**Table 5:** MANOVA analysis of the variables by weekly practice time and national member team.

	**National member team**				**F(df)**	***p*-value**	***$\eta_{p}^{2}$***
	**No**		**Yes**				
	**Weekly sport practice time**		**Weekly sport practice time**				
	**<10 h**	**10 h or more**	**<10 h**	**10 h or more**			
	**M (SD)**	**M (SD)**	**M (SD)**	**M (SD)**			
Optimism^*^	2.95 (0.6)	2.42 (0.4)	2.54 (0.8)	2.76 (0.6)	7.03 (1)	0.010	0.08
Anger^*^	1.02 (0.9)	1.59 (1.1)	1.44 (1.0)	1.07 (1.0)	4.26 (1)	0.042	0.05
Fatigue	1.24 (0.9)	1.28 (0.8)	1.25 (0.8)	1.14 (1.1)	0.11 (1)	0.743	0.00
Tension	1.71 (0.9)	2.09 (0.6)	2.10 (0.6)	1.83 (0.8)	2.94 (1)	0.090	0.04
Depression	0.61 (0.8)	0.73 (0.7)	0.85 (0.6)	0.69 (0.8)	0.58 (1)	0.449	0.01
Vigor	2.84 (0.5)	3.12 (0.5)	2.68 (0.6)	2.73 (0.8)	0.56 (1)	0.457	0.01
Emotional exhaustion	2.11 (0.7)	2,18 (0.8)	2.34 (0.8)	2.46 (0.9)	0.02 (1)	0.893	0.00
Despersonalization	1.76 (0.5)	1.87 (0.6)	1.82 (0.5)	2.12 (0.9)	0.40 (1)	0.527	0.01
Reduced efficacy	3.87 (0.6)	3.99 (0.6)	3.90 (0.6)	3.66 (0.7)	1.70 (1)	0.195	0.02

* *p ≤ 0.05. $\eta_{\text{p}}^{2}$, partial squared eta; $\eta_{\text{p}}^{2}$ < 0.01, trivial effect; 0.01 < $\eta_{\text{p}}^{2}$ < 0.06, small effect; 0.06 < $\eta_{\text{p}}^{2}$ < 0.14, medium effect; $\eta_{\text{p}}^{2}$ > 0.14, significant effect*.

## Discussion

According to the results, the general level of optimism of the kayak polo players was moderate, although the mood profile of the athletes did present a high value with medium levels of tension, fatigue, and anger and low levels of depression. The latter coincides with the findings of some studies, which found low levels of tension, depression, anger, and confusion for football players (Hassmen and Blomstrand, 1995), as well as with others, which found low levels of depression, confusion, and total mood disturbance for female basketball players (Henderson et al., 1998).

Inferential analysis showed that optimism was significantly related to the mood of anger and depression, as well as emotional exhaustion, consistent with findings from other research, where more optimistic individuals had smaller increases in negative mood and less reduction in mental vigor, suggesting the benefit of optimism in coping with potentially stressful situations (Brydon et al., 2009). Furthermore, other research showed that optimism had a significant negative relationship with both stress and burnout, with their mediation analyses indicating that perceived stress fully mediated the links between optimism, symptoms of emotional exhaustion, and sport devaluation, and also mediated the link between the optimism variable and a third symptom, the reduced sense of accomplishment. Thus, individual factors such as optimism may play a key role in the development of burnout by virtue of its association with stress (Gustafsson and Skoog, 2012; Gustafsson et al., 2018).

On the other hand, the correlation between mood and burnout was also significant, except for the relationship between the dimensions of tension and vigor. This was consistent with previous work, where perceived social support was generally associated with a lower degree of emotional exhaustion, whereas negative expectations of mood regulation were negatively correlated with burnout. Furthermore, negative expectations of mood regulation affected burnout more than social support. This finding suggests that burnout can be alleviated by controlling negative expectations of mood regulation through the intervention of training programs to improve such expectations (Kim et al., 2009).

In relation to the three levels of optimism that determined three groups depending on whether it was high, moderate, or lower, the second was the most numerous, with half of the canoeists in this group, followed by the first, although the number of kayak polo players was similar to the group with the lower level of optimism. Precisely, the two groups with moderate and high levels of optimism obtained a similar mood profile, with moderate-low scores in fatigue, as occurred in other previous studies where they showed that mental fatigue had a significant effect on endurance, but not for strength, probably due to the greater prolongation in time in terms of effort in the former compared with the latter, especially in collaborative-opposition sports such as kayak polo. Similarly, these authors showed that fatigue could be influenced by the level of optimism, understanding it as a coping sub-factor along with endurance and strength (Sook, 2018).

However, in addition, for these two groups, the values of tension and anger were also moderate–low, in line with other studies (Gustafsson and Skoog, 2012; Chirivella et al., 2013) and low depression, as occurred with the research carried out by other authors, where a positive relationship was found between optimism and sports performance in football players, concluding that when faced with a defeat, optimistic athletes faced the situation better than pessimists, with the difference in positive or negative optimism not being significant when the result was a victory. Furthermore, these analyses suggested that the negative relationships observed were attributed to negative outcomes due to lack of effort, known as defensive pessimism vs. lack of ability, which was defined as depressive pessimism (Gordon, 2008). The opposite was true for vigor, being the values high for these two groups of higher optimism. The findings obtained could be influenced by the fact that the high optimism group was composed of the most veteran players, half of whom had been international players at some point. This situation was associated with a higher competitive demand in which a higher level of performance is required, in the same way that those with moderate optimism, although less veteran, were also composed of players who had been international players at some point.

On the other hand, the low optimism group had a lower level of vigor, with moderate-high stress scores and a significant difference in the emotional exhaustion variable with respect to the high optimism group, probably because this third group is in backward positions and with purely defensive functions such as the goalkeeper, with a low achievement motivation; these results coincide with those obtained in other studies (Avugos et al., 2020).

## Conclusion

The current work provides information regarding the psychological profile of kayak polo players, where from the analysis of the three variables under study, canoeists who presented moderate levels of optimism also presented medium-low values in the different negative factors of mood. Likewise, the findings showed that optimism significantly influenced the emotional exhaustion of the athletes, with the level of stress to which the canoeist could be subjected playing an important role in the adjustment of these two variables. In addition, seniority and experience with international competition proved to be influential factors in the level of optimism achieved, as well as in the moderation of negative moods. These results suggest the importance and need to develop psychological profile training strategies to be incorporated into their work plans. Thus, it would be interesting to carry out future research involving a larger sample size in order to confirm and contrast the findings of the present study, as well as an experimental intervention proposal through a program or work plan for the psychological variables under study.

## Data Availability Statement

The raw data supporting the conclusions of this article will be made available by the authors, without undue reservation.

## Ethics Statement

The studies involving human participants were reviewed and approved by Comisión de Ética de Investigación–Universidad de Murcia. The patients/participants provided their written informed consent to participate in this study. Written informed consent was obtained from the individual(s) for the publication of any potentially identifiable images or data included in this article.

## Author Contributions

FB-B conceptualized the study. SA and LS-B defined the purpose of the study. SA and FO-M formulated the methodology. SA performed the formal analysis. LS-B performed the data collection and investigation. FO-M, VM-B, and FB-B edited and revised the manuscript. VM-B provided supervision. All authors contributed to the writing of the study and the article and approved the submitted version.

## Conflict of Interest

The authors declare that the research was conducted in the absence of any commercial or financial relationships that could be construed as a potential conflict of interest.
