# Supplementary material for: Mood, Burnout, and Dispositional Optimism in Kayak Polo Players During Their Competitive Stage
Source: Front Psychol. 2021 May 21;12:667603. doi: 10.3389/fpsyg.2021.667603 (PMC8175787; doi:10.3389/fpsyg.2021.667603)
Supplement: Supplementary file 1 [file Data_Sheet_1.docx]

Supplementary Material

# Supplementary Data

Angosto, Salvador; Salmeron-Baños, Laura; Montero, Ortín-Montero, Francisco José; Morales-Baños, Vicente; Borrego-Balsalobre, Francisco José (2021): Database kayak polo players. figshare. Dataset.

<https://doi.org/10.6084/m9.figshare.14339120.v1>

Angosto, Salvador; Salmeron-Baños, Laura; Montero, Ortín-Montero, Francisco José; Morales-Baños, Vicente; Borrego-Balsalobre, Francisco José (2021): Study information Study Kayak polo. figshare. Figure. Informed consent in initial information of the questionnaire.

<https://doi.org/10.6084/m9.figshare.14339108.v2>
